# Lived experience as systems-relevant evidence in psychedelic public health

**DOI:** 10.3389/fpubh.2026.1951513

**Published:** 2026-09-15

**Authors:** Pedram Dara

**Affiliations:** Psychedelic Lived Experiences, Ottawa, ON, Canada

**Keywords:** health governance, lived experience, lived experience expertise, patient safety, psychedelic clinical trials, psychedelic policy, psychedelic public health, psychedelic therapy

## Abstract

As psychedelic interventions move from controlled clinical trials into broader implementation, critical dimensions of lived experience among patients, trial participants, families, and caregivers remain systematically undermeasured in research, service design, and oversight. The resulting gap between protocol-defined outcomes and real-world experiences over time creates blind spots in current approaches, particularly regarding safety, care quality, post-treatment stabilization, aftercare, and long-term vulnerability. I argue that lived experience is a form of systems-relevant evidence essential to strengthening decision-making and institutional accountability. I propose a public health framework for integrating lived experience and expertise into psychedelic research, care models, and governance. Integrating these forms of experiential knowledge can enhance outcome visibility beyond standard clinical measures and adverse event reporting by identifying both beneficial changes and emerging risks across care pathways that might otherwise remain obscured. Finally, I outline key implications for program evaluation, workforce development, and policy design. A rigorous psychedelic public health strategy requires stronger mechanisms for understanding safety and long-term efficacy to ensure that implementation both optimizes outcomes and provides accountability and protection.

## Introduction

As psychedelic interventions transition from controlled research into clinical practice, questions about their population-level effects and governance needs are becoming increasingly important. Public health considerations extend beyond efficacy and acute adverse events to how patients experience the delivery and monitoring of care in various settings. Psychedelic public health is emerging as a field concerned with benefits, harms, and equity at community and population levels, yet engagement by public health researchers, institutions, and practitioners in the current psychedelic resurgence remains limited (1). Because long-term safety and durability remain incompletely characterized, I argue that broader implementation should include safeguards capable of monitoring outcomes as psychedelic interventions scale up (2). A recent meta-analysis reports promising efficacy signals, but methodological issues such as functional unblinding, expectancy and selection biases, and limited long-term follow-up complicate interpretation (3, 4). These limitations highlight the need for identifying which real-world outcomes matter and how they are lived.

Psychedelic interventions raise public health concerns because their effects can disrupt relational and social dynamics and are highly sensitive to context, suggestibility, support, and treatment environments (5). Outcomes are often shaped not only by pharmacological effects but also by expectations, preparation, therapeutic alliance, dosing conditions, integration, and social support post-treatment (5–7). An international group of psychedelic researchers, clinicians, and past trial participants found that reporting these contextual variables remains inconsistent (7). These contextual influences can contribute to both benefit and harm, making gaps between protocol-defined outcomes and real-world experiences over time particularly important. Existing research and oversight models often prioritize symptom change, treatment completion, and standard adverse event reporting. While these indicators are essential, they do not fully capture how patients and families experience benefits, risks, or longer-term changes in well-being after treatment.

This is partly an evidentiary problem. Clinical research often treats participant accounts as subjective and therefore of limited scientific value. When aggregated and systematically analyzed, these accounts can generate lived experience evidence that reveals protocol flaws, consent issues, and care pathway vulnerabilities. Excluding this knowledge produces epistemic injustice by treating people as sources of data rather than legitimate knowers and creators of medical evidence (8, 9). In addition, clinical research has traditionally devalued qualitative data, particularly first-person accounts of non-linear recovery or harm. Yet lived and living experience of a condition or intervention can identify needs, outcomes, and priorities for product development and health-system decision-making (10, 11). In psychedelic care, these forms of knowledge are vital because treatment effects can be highly unpredictable, context-dependent, and difficult to understand through conventional outcome measures alone (12). When captured, lived experience data can function as systems-relevant evidence: essential information that reveals how interventions are actually felt, where support structures fail, which risks remain hidden, and how real-world care diverges from protocol assumptions. I argue that lived experience must be treated as systems-relevant evidence within psychedelic public health, including across research, care, and governance.

## The measurement gap in psychedelic research

The efficacy of psychedelic interventions is often evaluated through symptom change on standardized clinical scales. Although such measures are necessary, they do not fully capture how patients and trial participants function in daily life or how families and caregivers experience the consequences of treatment over time. For example, progress in trials of MDMA-assisted therapy for PTSD has been assessed primarily through changes in the Clinician-Administered PTSD Scale for DSM-5 (13, 14), while participants’ actual experiences of improvement or deterioration may be shaped by factors such as changes in habits or relationships, capacity to work, shifts in worldviews, quality of support, or post-dosing difficulties (12, 15, 16). The result is a persistent gap between what is measured and what matters for implementation, accountability, and public health decision-making.

Safety measurement presents a similar problem. Trials record events such as headache, nausea, fatigue, and anxiety (17). FDA guidance recommends longer-term follow-up and documentation of session-related central nervous system effects, including hallucinations and euphoria. It also states that expected psychoactive effects should be recorded as adverse events even when participants do not describe them as adverse, including experiences characterized as positive, favorable, or neutral (2). This creates a classification tension because adverse event coding alone may not distinguish distress from therapeutic processing. Methods for identifying and reporting delayed emotional, relational, and situational difficulties remain underdeveloped and inconsistent (2, 17, 18). Recent evidence on extended difficulties includes a self-selected sample drawn largely from naturalistic, non-clinical use (15), underscoring a blind spot in formal clinical safety tracking. Safety may also involve post-treatment destabilization affecting mood, identity, beliefs, relationships, or coping after people leave structured care. Not all post-dosing difficulties signal harm. Changes may be beneficial, difficult, or both. Problems arise when systems assume that destabilization is always therapeutic, temporary, or self-resolving. Without adequate support, difficulties may intensify and harm patients (15, 16, 19). Experiences outside reporting windows or predefined categories may remain invisible (2, 17), revealing a mismatch between existing oversight and the ICH E6(R3) Good Clinical Practice guideline’s emphasis on proactive risk management, participant safety, and reliable trial results (20).

This measurement problem becomes especially apparent at transition points. Attempts to isolate pharmacological effects from accompanying psychological support may overlook how treatment context shapes outcomes and complicates safety assessment (2, 5). Although FDA guidance considers post-protocol care-seeking relevant to long-term follow-up (2), current models still undermeasure what happens when protocols end before stabilization. As formal support diminishes with treatment completion, emerging reports of extended difficulties, combined with the limited evidence base for psychedelic integration, suggest the need to examine discharge criteria, follow-up periods, referral pathways, and post-treatment support (6, 15, 16). These gaps affect how safety and implementation readiness are evaluated. From a public health perspective, what remains unmeasured remains ungoverned, leaving systems blind to how benefits and harms are actually lived.

## Why lived experience is not merely anecdotal

Within clinical research and psychiatric history, lived experience perspectives have often been treated as anecdotal and secondary to standard forms of evidence. Anecdotes are typically idiosyncratic, unsystematic, and difficult to interpret beyond the individual case, making them poorly suited to guide public health approaches on those terms alone. However, this limitation does not make lived experience inherently non-evidentiary. When collected systematically, analyzed for patterns, and linked to concrete decisions, lived experience can move past individual accounts and function as systems-relevant evidence (10, 12).

Expectancy and placebo effects are not unique to psychedelics, but their implications may be consequential when interventions deliberately combine drug effects with preparation, monitored dosing, psychological support, and integration (2, 5, 6). Placebo-controlled designs may also warrant ethical consideration when participants with serious mental health conditions face delays before potential access to investigational treatment. Standardized symptom scales and acute adverse event reporting do not always fully capture their safety or efficacy (12, 17, 18). Qualitative lived experience data can help identify how interventions are actually navigated, where support structures fail, how distress or instability emerges over time, and which beneficial changes or safety risks remain insufficiently visible within standard monitoring. Conventional outcome models may undercapture experiences at care transitions. Informal caregivers, including family members, partners, and close friends, may be among the first to observe post-treatment instability, impaired functioning, or support needs after formal oversight ends. Despite this, their perspectives have received limited attention in mental health research, service design, and adolescent psychedelic-assisted therapy (11, 21). Their exclusion narrows the evidence available to researchers, providers, and regulators, and can obscure signals about quality of care, real-world safety, and caregiver burden.

Treating lived experience as evidence can complement established scientific methods. First-Person Psychopharmacology (1PPP), for example, combines randomized trials with qualitative and idiographic methods to examine subjective drug effects within personal and social contexts (22). Quantitative outcomes may indicate whether change occurred, while structured qualitative evidence can reveal how it was experienced and sustained, where risks emerged, and which dimensions of recovery or harm remained unmeasured (10, 12). Lived experience expertise can inform person-centered services and identify practical gaps that protocol design and risk management may overlook. Together, these measurement and evidentiary gaps underscore the need to integrate lived experience into decision-making across psychedelic research, care, and policy.

## A framework for integrating lived experience into public health governance

I propose a framework that treats lived experience as systems-relevant evidence for shaping how psychedelic care is designed, delivered, and governed. The framework draws on published literature, my experience as a former participant in an MDMA-assisted therapy clinical trial, peer support work, and broader field engagement since 2019, including interviews conducted for the Psychedelic Lived Experiences Summit.^1^ Although these activities were not undertaken or systematically analyzed as research, they provided experiential and practice-based context that informed the framework’s three core pillars:

### An epistemic shift

Moving lived experience from a qualitative supplement to systems-relevant evidence for quality, safety, and governance that complements standardized clinical endpoints.

### A biopsychosocial lens

Recognizing that psychedelic outcomes are shaped by treatment context and by inequities in access to care and support based on personal, social, cultural, and structural factors, which may influence who benefits, who experiences harm, and whose outcomes become visible within research and care systems (1, 5). This emphasis on drug-induced experience and context is compatible with Moncrieff’s drug-centered model, which contrasts with models that assume therapeutic effects arise solely from correcting an underlying disease process (23).

### Design-based governance

Treating governance as a proactive design discipline that shapes safer systems from the outset. Lived experience data should directly inform protocol design, consent processes, discharge criteria, aftercare, escalation pathways, workforce training, and mechanisms for institutional and public accountability.

Under this framework, lived experience evidence can inform decision-making across five interconnected domains:

### Outcome visibility beyond conventional reporting

Current outcome measures may undercapture positive changes that people consider central to recovery, such as restored agency, renewed purpose, improved relationships, increased self-compassion, or improved quality of life (12). These outcomes may extend beyond diagnosis-specific symptom reduction and remain invisible unless they are examined through longitudinal and qualitative methods. The same measurement gap applies to risk. Conventional adverse event monitoring remains essential, but definitions and reporting of delayed, context-dependent distress vary across trials and between registries and publications, limiting comparison and longer-term risk characterization (17, 18, 24). A testable concern is that transference, gratitude, fear of disappointing the care team, or concern about affecting study findings may inhibit disclosure of destabilization or boundary concerns. Independent, peer-informed reporting channels should therefore be evaluated for reducing disclosure hesitancy and identifying harms or support failures missed by standard monitoring.

### Discharge and transition quality

Risk visibility becomes critical at discharge, when clinical protocols may end before some participants feel psychologically, relationally, or practically stabilized. FDA guidance recognizes increased vulnerability and suggestibility during treatment and recommends quantitative and qualitative assessment of drug effects to inform discharge safety (2). Complementary person-centered recovery frameworks identify trust, confidence, and access to basic needs and resources as foundational domains prioritized by service users (25). Psychedelic programs should test whether these domains and clear escalation pathways improve discharge-readiness assessment and care transitions.

### Aftercare and continuity of support

Psychedelic integration is generally understood as an ongoing process of interpreting and incorporating an experience into daily life, but its definitions, practices, and evidence base remain inconsistent (6). Participants may move abruptly from highly structured dosing phases into limited or absent aftercare, creating a discontinuity in long-term care (6, 15, 16). Emerging research on extended difficulties indicates that some individuals continue to require support well after the acute drug experience (15, 16, 19). Longitudinal lived experience data should test whether integration and referral systems meet support needs and identify unrecognized family and caregiver burdens. Where follow-up is limited to instructions to seek emergency services, support remains reactive. A lived experience-informed model embeds routine follow-up and peer support before crises develop. Aftercare should be built into the intervention journey and funded well past dosing. This framework therefore proposes that payment, reimbursement, and regulatory arrangements support preparation, integration, follow-up, and referral, while assigning clear responsibility for continuity of care rather than treating drug administration as an isolated event.

### Workforce and service design

Delivering high-quality psychedelic care requires a workforce equipped to recognize and respond to profound psychological shifts, including ontological shock, an abrupt destabilization of core assumptions about self and reality that may have negative, positive, or mixed consequences not captured by standard clinical measures (19). Extended difficulties, including heightened anxiety, social disconnection, and existential struggle, may persist for months or years and may benefit from professional, peer, or family support (26). This raises a key, unresolved governance question: how can services distinguish adverse events requiring clinical intervention from distressing but non-pathological transitions requiring specialized aftercare? Interprofessional teams should collaborate with lived experience experts to improve recognition and interpretation of such nuanced experiences (11, 27, 28). This approach may help avoid misinterpreting such experiences as clinical deterioration or labeling struggling individuals as non-responders. Because integration occurs within relational ecosystems, service design and lived experience-informed metrics need to extend beyond the individual to relational health, post-treatment isolation, and family burden. Proactive preparation is therefore needed for families and caregivers facing sudden positive or negative shifts in identity, emotion, worldview, and priorities so that relational dynamics can support integration, not hinder it (21). Finally, methods such as participatory action research and co-design, which position affected stakeholders as partners in innovation, can identify blind spots across the care pathway, reduce tokenism through iterative co-creation, and support services and training aligned with patient needs (11, 29, 30).

### Program evaluation and policy feedback

Ultimately, what gets measured shapes what institutions can learn and improve. Evaluating programs solely through symptom scores, protocol completion, and standard tools keeps health systems blind to real-world functioning. Lived experience data should enhance evaluation by identifying discrepancies between formal endpoints and long-term outcomes, mitigating risk, and feeding critical signals back to clinicians, sponsors, and regulators. The framework is intended to strengthen high-stakes decisions from protocol design and screening through discharge, follow-up, and regulatory review. Lived experience data can be captured through qualitative interviews and focus groups, longitudinal or mixed-method studies, caregiver input, participatory action research, and formal advisory bodies such as patient councils or lived experience boards (10–12, 15, 31). The resulting evidence should then reach decision-making bodies, including ethics committees, oversight boards, sponsors, and regulators (10, 11, 31). But if the signal never reaches a decision point, it cannot improve quality or safety. The practical test is whether information changes protocol design, discharge criteria, training standards, or regulatory requirements. Vital aspects of ethics, safety, support, and accountability remain difficult to govern without such data, making the integration of lived experience essential for rigorous implementation.

## Discussion

Shifting lived experience from an anecdotal supplement to systems-relevant evidence requires clinical, practical, and regulatory frameworks to expand what counts as evidence for safety, efficacy, and implementation readiness. A better program design accounts for dosing and discharge readiness, post-protocol destabilization, long-term follow-up, and the experiences of families and caregivers at key transition points. This calls for a more interdisciplinary approach to psychedelic public health that invests in the care infrastructure surrounding the intervention and integrates clinical science, qualitative inquiry, health services research, implementation science, and lived experience expertise into shared decision-making.

When systematically collected and analyzed, recurring experiences across people, programs, and settings can generate system-level public health signals. However, both positive effects and difficult experiences continue to be undercaptured, limiting understanding of how benefits and risks are experienced and sustained beyond symptom changes. As psychedelic interventions move from controlled trials into real-world care, community-based support matters because risks and benefits do not end when protocols end (12, 15, 16, 26). Equity and context also shape outcome visibility: differences in access to care and support, and in the settings surrounding treatment, may influence who benefits, who experiences harm, and which experiences enter institutional decision-making (1, 5).

I identify three systemic risks that may threaten the inclusion of lived experience in psychedelic governance. The first risk is tokenism, which confines patients or survivors to low-level advisory roles without genuine influence over decisions (29). Ethical governance in a rapidly scaling field requires diverse perspectives, including those with relevant lived experience (32). The second risk is co-optation, whereby institutions, commercial actors, media, or advocacy groups selectively amplify or marginalize lived experience accounts to support preferred narratives. This concern is informed by scholarship on epistemic injustice and psychedelic medicalization but requires further empirical study (8, 33). The third risk is epistemic friction, which occurs when experiential knowledge faces biomedical hierarchies and is dismissed as anecdotal (8, 9). Mitigating these risks requires ethical leadership, transparent selection, methodological rigor, shared decision-making authority, and traceable feedback loops. This aligns with WHO Europe’s recent roadmap for the meaningful integration of lived experience practitioners across mental health policy, services, workforce, and community settings (31).

The proposed framework has not been empirically validated and requires testing across settings, including whether independent reporting improves disclosure, lived experience-informed discharge assessment identifies support needs, structured follow-up improves continuity, and feedback produces documented protocol or policy changes. Lived experience data can be inconsistently captured, difficult to standardize, and vulnerable to selective interpretation if handled carelessly. Its integration into regulatory frameworks may create methodological and institutional challenges. Because psychedelic interventions are complex, experiential knowledge should complement quantitative outcomes, randomized evidence, and conventional safety monitoring. Otherwise, current models of research and care remain incomplete. This matters most at moments when risks, support failure, and long-term consequences are least visible, and when positive gains extend beyond standardized clinical measures.

Psychedelic interventions are advancing under conditions of genuine promise and unresolved uncertainty. For example, the FDA’s 2024 decision not to approve MDMA for PTSD highlighted concerns about safety data reliability, including the reporting of adverse events characterized as positive or favorable, as well as treatment durability, expectancy bias, and functional unblinding (34). Decisions made during this period of early implementation may shape the governance of psychedelic medicine for a generation. The field’s rapid expansion must be matched by scientific rigor, adequate safeguards, and ethical institutions willing to examine their decisions. As an emerging discipline, psychedelic public health will require more than evidence of efficacy under controlled conditions. It will require systems capable of learning from how interventions are actually lived across care pathways and beyond, including outcomes that remain invisible within conventional measures. Stronger mechanisms are needed to capture, interpret, and integrate lived experience into governance, service design, evaluation, and policy. Otherwise, implementation may look successful on paper while risk, inequity, and unmet needs remain insufficiently recognized. Integrating lived experience into psychedelic public health decision-making is therefore essential to surface systemic blind spots and build systems of long-term care that are safer, more effective, more inclusive, and more accountable.

## Data Availability

The original contributions presented in the study are included in the article/supplementary material, further inquiries can be directed to the corresponding author.

## Footnotes

1 Psychedelic Lived Experiences. Psychedelic Lived Experiences Summit. Available at: https://psychedeliclivedexperiences.com/summit (Accessed July 24, 2026).

## References

[1] Kuiper H Alley C Harris Z Kuiper Rauch C Robbins M Rodriguez P . Psychedelic public health: state of the field and implications for equity. Soc Sci Med. (2024) 357:117134. doi: 10.1016/j.socscimed.2024.117134, 39173415

[2] US Food and Drug Administration. Psychedelic drugs: considerations for clinical investigations. Guidance for industry (2026). Available online at: https://www.fda.gov/media/169694/download (Accessed July 23, 2026).

[3] Yao Y Guo D Lu TS Liu FL Huang SH Diao MQ . Efficacy and safety of psychedelics for the treatment of mental disorders: a systematic review and meta-analysis. Psychiatry Res. (2024) 335:115886. doi: 10.1016/j.psychres.2024.115886, 38574699

[4] van Elk M Fried EI. History repeating: guidelines to address common problems in psychedelic science. Ther Adv Psychopharmacol. (2023) 13:20451253231198466. doi: 10.1177/20451253231198466, 37766730 PMC10521293

[5] Carhart-Harris RL Roseman L Haijen E Erritzoe D Watts R Branchi I . Psychedelics and the essential importance of context. J Psychopharmacol. (2018) 32:725–31. doi: 10.1177/0269881118754710, 29446697

[6] Bathje GJ Majeski E Kudowor M. Psychedelic integration: an analysis of the concept and its practice. Front Psychol. (2022) 13:824077. doi: 10.3389/fpsyg.2022.824077, 35992410 PMC9386447

[7] Pronovost-Morgan C Greenway KT Roseman L The ReSPCT experts. An international Delphi consensus for reporting of setting in psychedelic clinical trials. Nat Med. (2025) 31:2186–95. doi: 10.1038/s41591-025-03685-9, 40461819 PMC12283393

[8] Okoroji C Mackay T Robotham D Beckford D Pinfold V. Epistemic injustice and mental health research: a pragmatic approach to working with lived experience expertise. Front Psych. (2023) 14:1114725. doi: 10.3389/fpsyt.2023.1114725, 37056406 PMC10086175

[9] Carel H Kidd IJ. Epistemic injustice in healthcare: a philosophical analysis. Med Health Care Philos. (2014) 17:529–40. doi: 10.1007/s11019-014-9560-2, 24740808

[10] US Food and Drug Administration. FDA Patient-Focused Drug Development Guidance Series for Enhancing the Incorporation of the Patient’s Voice in Medical Product Development and Regulatory Decision Making (2025). Available online at: https://www.fda.gov/drugs/development-approval-process-drugs/fda-patient-focused-drug-development-guidance-series-enhancing-incorporation-patients-voice-medical (Accessed July 23, 2026).

[11] World Health Organization. WHO Framework for Meaningful Engagement of People Living with Noncommunicable Diseases, and Mental Health and Neurological Conditions. Geneva: World Health Organization (2023). Available online at: https://www.who.int/publications/i/item/9789240073074 (Accessed July 23, 2026).

[12] Breeksema JJ Niemeijer AR Krediet E Vermetten E Schoevers RA. Psychedelic treatments for psychiatric disorders: a systematic review and thematic synthesis of patient experiences in qualitative studies. CNS Drugs. (2020) 34:925–46. doi: 10.1007/s40263-020-00748-y, 32803732 PMC7447679

[13] Mitchell JM Bogenschutz M Lilienstein A Harrison C Kleiman S Parker-Guilbert K . MDMA-assisted therapy for severe PTSD: a randomized, double-blind, placebo-controlled phase 3 study. Nat Med. (2021) 27:1025–33. doi: 10.1038/s41591-021-01336-3, 33972795 PMC8205851

[14] Mitchell JM Ot’alora GM van der Kolk B Shannon S Bogenschutz M Gelfand Y . MDMA-assisted therapy for moderate to severe PTSD: a randomized, placebo-controlled phase 3 trial. Nat Med. (2023) 29:2473–80. doi: 10.1038/s41591-023-02565-4 PMC10579091 37709999

[15] Evans J Robinson OC Argyri EK Suseelan S Murphy-Beiner A McAlpine R . Extended difficulties following the use of psychedelic drugs: a mixed methods study. PLoS One. (2023) 18:e0293349. doi: 10.1371/journal.pone.0293349, 37874826 PMC10597511

[16] Bremler R Katati N Shergill P Erritzoe D Carhart-Harris RL. Case analysis of long-term negative psychological responses to psychedelics. Sci Rep. (2023) 13:15998. doi: 10.1038/s41598-023-41145-x, 37749109 PMC10519946

[17] Breeksema JJ Kuin BW Kamphuis J van den Brink W Vermetten E Schoevers RA. Adverse events in clinical treatments with serotonergic psychedelics and MDMA: a mixed-methods systematic review. J Psychopharmacol. (2022) 36:1100–17. doi: 10.1177/02698811221116926, 36017784 PMC9548934

[18] Marinis J Clarke ST Guerin AA Guastella AJ Bedi G. Reporting of side-effects in clinical trials of psilocybin-assisted psychotherapy for psychiatric conditions: systematic review. BJPsych Open. (2025) 11:e261. doi: 10.1192/bjo.2025.10847, 41178084 PMC12641409

[19] Argyri EK Evans J Luke D Michael P Michelle K Rohani-Shukla C . Navigating groundlessness: an interview study on dealing with ontological shock and existential distress following psychedelic experiences. PLoS One. (2025) 20:e0322501. doi: 10.1371/journal.pone.0322501, 40323979 PMC12052184

[20] International Council for Harmonisation of Technical Requirements for Pharmaceuticals for Human Use. ICH Harmonised Guideline: Guideline for Good Clinical Practice E6(R3). Final Version (2026). Available online at: https://database.ich.org/sites/default/files/ICH%20E6%28R3%29_Step4_FinalConsolidatedGuideline_2026_0616_.pdf (Accessed July 25, 2026).

[21] Forsberg S Downey AE Bruett LD Hail L Fernandes-Osterhold G Woolley JD . Leveraging family and caregiver support in psychedelic-assisted therapy: considerations for the treatment of adolescents. Child Adolesc Psychiatry Ment Health. (2025) 19:64. doi: 10.1186/s13034-025-00930-4, 40468435 PMC12139335

[22] Stupak R Lichtenberg P. First-person psychopharmacology (1PPP): a new way forward for clinical trials. J Humanist Psychol. (2026). doi: 10.1177/00221678261455500

[23] Moncrieff J. Research on a ‘drug-centred’ approach to psychiatric drug treatment: assessing the impact of mental and behavioural alterations produced by psychiatric drugs. Epidemiol Psychiatr Sci. (2018) 27:133–40. doi: 10.1017/S2045796017000555, 29022518 PMC6998955

[24] Žuljević MF Mijatović A Orhanović R Goasdoué G Gujinović D. Consistency of protocol and safety data reporting in clinical trial registrations and corresponding publications of interventions involving MDMA and psilocybin. J Clin Epidemiol. (2026) 192:112170. doi: 10.1016/j.jclinepi.2026.112170, 41581835

[25] Seidman J Hand R Williams G Rice K Embedding Measures that Matter into Mental Health Systems: a Lived Experience-Informed Measurement Framework for Serious Mental Illness and Guidance for Future Initiatives New York, NY Fountain House (2025). Available online at: https://www.fountainhouse.org/assets/FH_MTM_Report_R05.pdf (Accessed July 23, 2026).

[26] Robinson OC Evans J McAlpine RG Argyri EK Luke D. An investigation into the varieties of extended difficulties following psychedelic drug use: duration, severity and helpful coping strategies. J Psychedelic Stud. (2025) 9:117–25. doi: 10.1556/2054.2024.00420

[27] World Health Organization. Framework for Action on Interprofessional Education and Collaborative Practice. Geneva: World Health Organization (2010). Available online at: https://www.who.int/publications/i/item/framework-for-action-on-interprofessional-education-collaborative-practice (Accessed July 23, 2026).

[28] Viking T Wenzer J Hylin U Nilsson L. Peer support workers’ role and expertise and interprofessional learning in mental health care: a scoping review. J Interprof Care. (2022) 36:828–38. doi: 10.1080/13561820.2021.2014796, 35129027

[29] Ocloo J Matthews R. From tokenism to empowerment: progressing patient and public involvement in healthcare improvement. BMJ Qual Saf. (2016) 25:626–32. doi: 10.1136/bmjqs-2015-004839, 26993640 PMC4975844

[30] Cornwall A Jewkes R. What is participatory research? Soc Sci Med. (1995) 41:1667–76. doi: 10.1016/0277-9536(95)00127-S, 8746866

[31] World Health Organization Regional Office for Europe. Transforming Mental Health Through Lived Experience: Roadmap for Integrating Lived and Living Experience Practitioners Into Policy, Services and Community. Copenhagen: WHO Regional Office for Europe (2025). Available online at: https://www.who.int/europe/publications/i/item/WHO-EURO-2025-12307-52079-79927 (Accessed August 17, 2026).

[32] Jacobs E Earp BD Appelbaum PS Bruce L Cassidy K Celidwen Y . The Hopkins-Oxford psychedelics ethics (HOPE) working group consensus statement. Am J Bioeth. (2024) 24:6–12. doi: 10.1080/15265161.2024.2342764, 38695382

[33] Noorani T. Making psychedelics into medicines: the politics and paradoxes of medicalization. J Psychedelic Stud. (2020) 4:34–9. doi: 10.1556/2054.2019.018

[34] US Food and Drug Administration. Complete Response, NDA 215455 (2024). Available online at: https://download.open.fda.gov/crl/CRL_NDA215455_20240808.pdf (Accessed August 17, 2026).

